# Physicochemical properties, fermentative profile, microbiological quality, and pathogen contamination of baled silage during long‐term storage

**DOI:** 10.1002/jsfa.70807

**Published:** 2026-06-25

**Authors:** Lidia Nieddu, Mirko Fresu, Carmelo Mastroeni, Antonio Gallo, Severino Zara, Francesco Fancello, Alberto Stanislao Atzori

**Affiliations:** ^1^ Department of Agricultural Sciences University of Sassari Sassari Italy; ^2^ University School for Advanced Studies IUSS Pavia Pavia Italy; ^3^ Department of Animal Science, Food and Nutrition Università Cattolica del Sacro Cuore Piacenza Italy; ^4^ Romeo and Enrica Invernizzi Research Center for Sustainable Dairy Production of the Università Cattolica del Sacro Cuore (CREI) Piacenza Italy; ^5^ Institute for Animal Production System in Mediterranean Environment, National Research Council (CNR – ISPAAM) Sassari Italy

**Keywords:** 2‐propanediol, ammonia nitrogen, clostridia spores, *Listeria monocytogenes*, microbiological safety

## Abstract

**BACKGROUND:**

Baled silage is expanding in Mediterranean regions due to climate change and demand for high‐quality forage; therefore, evaluating the effects of long‐term storage on microbiological and fermentative quality is essential.

**RESULTS:**

In this study, 115 baled silages were sampled from different farms at 30, 60, 90, 180, and 270 days after baling. Chemical composition, fermentative compounds, microbial groups, and the presence of pathogens were assessed. The ensiling period did not affect any chemical parameter measured. Low concentrations of ammonia nitrogen (33.56 ± 27.33 g kg^−1^ total Kjeldahl nitrogen) were observed, and butyric acid was detected in only five samples, with a mean concentration of 2.11 ± 0.46 g kg^−1^. Lactic and acetic acids averaged 35 ± 1.99 and 15 ± 1.05 g kg^−1^ dry matter, respectively, with lactic acid decreasing and acetic acid increasing over time. Lactic acid bacteria were found at optimal concentrations (5.42 ± 2.50 log_10_ CFU g^−1^), contributing an average pH of 4.28 ± 0.48. Clostridia were absent in 73% of samples, and when detected ranged from 1.69 to 3.46 log_10_ CFU g^−1^. Fungi were found in 22% of samples (ranging from 2 to 6.19 log_10_ CFU g^−1^). Neither *Escherichia coli* nor *Listeria monocytogenes* was detected, indicating correct application of silage production techniques.

**CONCLUSION:**

After 270 days of storage, baled silage preserved its chemical–physical and microbial quality under Mediterranean farming conditions. pH remained stable, while lactic acid declined and acetate/1,2‐propanediol increased over time. Fungi and clostridia were mostly undetectable; *E. coli* and *L. monocytogenes* were not detected. © 2026 The Author(s). *Journal of the Science of Food and Agriculture* published by John Wiley & Sons Ltd on behalf of Society of Chemical Industry.

## INTRODUCTION

Ensiling forage in round bales is mainly practiced in farms with small herds since it allows reduction of losses and the production of high‐quality silage.^1^ Several recommendations such as harvesting at an appropriate dry matter (DM) concentration (e.g., 450–550 g kg^−1^ DM), setting the chopping length (10–40 mm) related to the crop, wrapping as soon as the forage is baled in the field, and applying a minimum of six layers of barrier plastic film should be considered to create an anaerobic environment that can prevent the development of spoilage microorganisms, and ensure effective fermentation.^2^

In Mediterranean dairy sheep systems, forage production is inherently shaped by climatic seasonality, with herbage growth concentrated in autumn and spring, and markedly reduced during the dry summer period. In these semi‐extensive systems, the quality of conserved forages is particularly important, especially where milk is used to produce Protected Designation of Origin (PDO) cheeses, since feed characteristics can affect milk composition and, consequently, the sensory properties, typicity, and technological aptitude of the final products. Within this framework, the production of baled silage allows one to anticipate grass cutting at an early vegetative stage of maturity (late winter early spring) and harvesting of haylages with high nutritional value compared to late‐maturity harvested hays conventionally produced at lower latitudes. Sardinia is included among the world leaders in the sheep cheese trade, with 13% of European sheep milk.^3^ In recent years, the use of baled silage in dairy sheep feeding in Sardinia and other Mediterranean countries (Atzori, personal communication) has become increasingly popular among small and medium‐sized farms. Producers find it attractive compared with other forage production methods for several reasons. Compared with hay, key advantages include shorter harvesting time, which enables earlier cuts with lower weather risk and yields forages with higher nutritional value. From a management perspective, the relatively simple mechanization required makes adoption feasible even in resource‐limited small and medium‐sized farms. Despite these benefits, some challenges remain for its acceptance along the dairy supply chain, and specifically in areas where PDO cheeses are produced. In particular, many dairy processors still exclude milk from animals fed silage and baled silage due to the potential presence of pathogenic and spoilage microorganisms that can compromise the cheesemaking process. The main issues reported include late cheese swelling caused by clostridia, early swelling associated with coliform presence, and contamination by *Listeria monocytogenes*. Moreover, alongside traditional baled silage, the ensiling of agricultural by‐products is increasingly adopted, providing local feed for livestock, reducing reliance on imported feed, and contributing to the circular economy by minimizing waste and disposal costs.^4^ The conservation process of baled silage is conceptually similar to that of conventional silage, as it is based on an anaerobic fermentation process aimed at preserving the nutritional value of fresh forage.^5^ However, several studies have shown that the chemical and microbiological composition of baled silage often differs from that of silages produced using conventional systems.^6^, ^7^ For instance, baled silage tends to exhibit a higher pH, indicative of a different fermentative environment and a different combination of metabolites compared to bunker silages. This condition may be less effective in inhibiting the growth of undesirable microorganisms, such as yeasts and *Clostridium*, potentially compromising the aerobic stability and hygienic quality of the silage.^8^ The microbial communities present on forage crops at harvest differ significantly from those that develop during the fermentation process and the feed‐out phase. This variation is primarily attributed to the differing tolerance and adaptability of microorganisms to the environmental conditions characterizing each stage of the conservation process.^9^ The fermentation process and safety of silage can be compromised by the presence of spoilage and pathogenic microorganisms such as yeasts, molds, enterobacteria, *Clostridium* spp., *Listeria monocytogenes*, *Escherichia coli*, *Bacillus* spp. and others that can deteriorate/contaminate the ensiled mass, affecting its hygienic and nutritional quality, and impair animal performance and derived products thereof.^10^ Currently, the scientific literature on baled silage remains relatively limited compared to the extensive body of research dedicated to chopped silage, particularly regarding fermentation‐related aspects. While most studies focus on forages preserved through conventional ensiling methods, the specific fermentation dynamics of baled silage, typically characterized by a higher dry matter content, are still not fully understood.

The aim of this study was to investigate the effects of long‐term storage on the presence and dynamics of the main microbial groups in wrapped haylage, with the objectives of increasing the understanding of the baled silage production and conservation, reducing microbiological and health risks; improving the hygienic quality of the final product; and promoting broader adoption of this preservation technique. The research also focuses on identifying and monitoring the presence of pathogens and spoilage bacteria relevant to public and animal health, including *Listeria* spp., *Escherichia coli* and *Clostridium* spp., analyzing their distribution and the risk factors associated with haylage production and conservation practices. Moreover, this study represents an initial step in assessing baled silage risks and impact on sheep farming systems. It may also serve as a case study for other dairy contexts where silage is not yet commonly used, particularly with regard to its potential effects on cheese quality.

## MATERIAL AND METHODS

### Baled silage sampling scheme

A total of 115 fermented products belonging to different botanical types (i.e., 19 pure grasses (G), 20 legumes (L), 25 grass mixes (MG), and 51 mixes of grasses and legumes (MGL), were collected from 15 different farms around Sardinia (Italy). These baled silages were sampled after 30, 60, 90, 180, and 270 days of ensiling. In all sampled farms, baled silages were treated under field conditions with commercial inoculants. Two main inoculant formulations were used: one based on *Lentilactobacillus buchneri* plus *Lactiplantibacillus plantarum*, and one based on *Lentilactobacillus buchneri* plus *Lactococcus lactis* and *Pediococcus pentosaceus*. Because inoculant formulation was not included as an experimental factor in the study, its possible effect on fermentation outcomes could not be evaluated separately from other farm‐related sources of variation. This aspect is therefore acknowledged as a limitation of the study.

Furthermore, information on forage botanical composition and number of plastic film layers was available and is reported in the Supporting Information (Table S1). More detailed data on bale density, chopping length, and other management‐related variables were not consistently available for all farms and were therefore not included in the statistical analysis. To account for farm‐specific heterogeneity, farm was included as a random effect in the mixed‐effects model (see statistical analysis section).

The sampling of the baled silage was performed using a toothed core cutter (45 mm diameter, 600 mm length), designed and fabricated in‐house, specifically developed to penetrate the closed bales and obtain a clean and homogeneous cut of the ensiled mass. Sampling was performed by fully inserting the core cutter in a position perpendicular to the side surface of the bale, orienting it towards the core of the bale. Three cores were taken from each bale. This procedure made it possible to take a representative portion of the ensiled material. The aliquots were carefully mixed in order to obtain a representative sample (~1 kg). Because the three subsamples collected from each bale were pooled into a single composite sample, within‐bale variability could not be estimated; this should be considered a limitation of the study.

### Chemical analyses

Samples from the first aliquot were dried at 60 °C in a forced‐air oven for 48 h, ground using a laboratory mill (Thomas‐Wiley, Arthur H Thomas Co., Philadelphia, PA, USA), and passed through a 1 mm mesh screen. The ground material was stored under appropriate conditions for subsequent chemical analysis. Dry matter (DM) content was determined by gravimetric measurement of the mass loss after drying at 105 °C for 3 h, according to AOAC (1995; method 945.15).^11^ Corrections for volatile compound losses during oven drying were applied using the equations proposed by NorFor.^12^ The ground samples were analyzed using a near‐infrared reflectance spectroscopy (NIRS) system (DS3, Foss, Hillerød, Denmark), equipped with a monochromator and a rotating cup module. Spectral data were collected over the 400–2500 nm wavelength range at 0.5 nm intervals. Prediction equations were used to calculate concentrations of crude protein (CP), ether extract (EE), ash, neutral detergent fiber (NDF), acid detergent fiber (ADF), acid detergent lignin (ADL), and starch. The calibrations used to obtain forage characterization were developed by internal laboratory of the Department of Animal Science, Food and Nutrition (DIANA), Università Cattolica del Sacro Cuore (Piacenza, Italy), based on the following wet chemistry assays. Calibrations equations for NIRS analysis (Supporting Information, Table S2) referred to CP, EE, ash, NDF, ADF, ADL, and starch were based on procedures described in AOAC method 990.03 (AOAC International, 2012), AOAC method 920.29, AOAC 942.05 (AOAC International, 2012), AOAC 2002.04 (AOAC, 2016), Hall (2015), Möller (2009), and AOAC method 920.39 (AOAC International, 2012), respectively.^11^

### Fermentative analyses

To quantify volatile organic acids, lactic acid, ethanol, and other alcohols, aldehydes, esters, and ketones, a wet sample (~50 g) was taken from a thawed aliquot and homogenized using a Stomacher blender (Seward Ltd, Worthing, UK) for 3 min in distilled water at a water‐to‐fresh weight ratio of 3:1.^13^ The mixture was filtered through gauze, and an aliquot was taken for pH measurement. To remove solid impurities, the mixtures were centrifuged at 4500 × *g* for 15 min at room temperature, and the liquid phase was retained for the determination of volatile fatty acids (VFA) and volatile organic compounds (VOC) using a gas chromatography (GC) system equipped with a flame ionization detector. GC parameters as well as identification and quantification of VFA and VOC were performed as previously described.^13^ Lactic acid was determined by high‐performance liquid chromatography (HPLC).^14^ When a compound was undetectable, its detection limit was reported as described.^13^ Ammonia nitrogen (ammonia N) concentration was determined on approximately 20 g of fresh sample in a slurry composed of 150 mL distilled water and magnesium oxide (10 g per sample).^12^ Ammonia N concentration was expressed as a fraction of total Kjeldahl nitrogen (TKN), and was determined after steam distillation using the Kjeldahl method (method 984.13).^11^ The pH measurement was carried out in the laboratory at sample arrival, before freezing, using a calibrated pH meter, following a standardized procedure consisting of the following steps: a 10 g portion of the feed was taken from the sampled mass and placed in a sterile stomacher bag; deionized water was added to reach a final weight of 100 g. The sample was then homogenized for 1 min using a Stomacher 400 Circulator (Seward Ltd). Upon completion of homogenization, the pH was measured directly on the resulting suspension after 30 min.

### Microbiological analyses

For microbiological analysis, 20 g of sample was weighed and placed inside a stomacher bag (1627 mL; Whirl‐Pak, Filtration Group, Austin, TX, USA) to which 180 mL buffered peptone water was added. Homogenization of the sample was performed for 4 min in a stomacher (Stomacher 400 Circulator). After this step, serial dilutions (10^−1^ to 10^−7^) were set up and used for seeding the culture media for the detection and count of the following microbial groups: lactic acid bacteria (LAB); total mesophilic count (TMC); yeasts and molds (Y&M); clostridia; aerobic spore‐formers bacteria (ASFB); and *Escherichia coli*. LAB counts were performed on MRS agar medium incubated at 25 °C under anaerobic conditions for 7 days. TMCs were performed on TSA (Tryptone Soy Agar) incubated at 25 °C under aerobic conditions for 7 days.

Y&M counts were performed on Rose Bengal Agar medium incubated at 25 °C for 5 days. *Escherichia coli* β‐glucuronidase positive counts were performed on TBX medium incubated at 44 °C for 24 h. For clostridia and ASFB detection, dilutions were heat‐treated at 80 °C for 10 min to remove all vegetative forms. Then, for the enumeration of clostridia, they were seeded on Reinforced Clostridial Medium (RCM) enriched with 200 mg L^−1^ cycloserine and 0.005% Neutral Red.^15^ On this medium, the typical clostridia colony formed yellow‐phosphorescent colonies under ultraviolet exposure. The medium was incubated at 36 °C for 7 days, after which typical colony counts were performed, while ASFB counts were performed on Brain Heart Infusion (BHI) incubated at 25 °C for 5 days.

The ISO 11290‐1 and ISO 11290‐2 methods were used for the determination and enumeration of *Listeria*, respectively.

To determine the species affiliation, the following protocol was followed. Presumptive *Listeria monocytogenes* taken from ALOA medium were isolated and purified, and DNA was extracted using Quick‐DNA Fungal/Bacterial Kits (Zymo Research, Irvine, CA, USA), following the manufacturer's instructions.

The extracted DNA was used to confirm the *Listeria monocytogenes* species membership of the isolates by species‐specific polymerase chain reaction (PCR), according to the protocol described,^16^ which amplifies a 730 bp region of the hlyA gene, coding for listeriolysin O (LLO) synthesis.

### Statistical analysis

LAB, TMC, and ASFB counts were log_10_‐transformed prior to statistical analysis, and the analysis of variance terms (standard errors of the means and *P*‐values) refer to these log_10_‐transformed values. For chemical and fermentative parameters, data distribution was checked before analysis to verify compliance with model assumptions. All variables were approximately normally distributed, except ammonia N, which was square‐root transformed prior to analysis. The data were analyzed using the linear mixed procedure using SPSS Statistics 22 software (IBM, Armonk, NY, USA) with the following model:

$$Y_{\mathrm{ijk}}=\mu +A_{i}+B_{j}+{\left(A\times B\right)}_{\mathrm{ij}}+C_{k}+e_{\mathrm{ijk}}$$

where *Y*_*ijk*_ is the dependent variable; *μ* is the overall mean; *A*_*i*_ represents the fixed effect of ensiling period; *B*_*j*_ is the fixed effect of the type of silage; (*A* × *B*)_*ij*_ denotes the interaction between factors *A* and *B*; *C*_*k*_ is the random effect of the farm; and *e*_*ijk*_ is the residual error.

When the interaction term was not significant, significant main effects were further explored by pairwise comparisons of estimated marginal means.

When the interaction term was significant (*P* < 0.05), the data were presented in the corresponding figure to illustrate the interaction between ensiled forage (EF) and ensiling period (EP), and pairwise comparisons of estimated marginal means were subsequently performed within each level of the interacting factors.

Because of the high frequency of non‐detects in Y&M and clostridia counts, censored‐data methods were used throughout, avoiding substitution of values below the detection limit. In accordance with established recommendations, substitution‐based approaches (e.g., zero, LOD or LOD/2) were deliberately avoided, as they may distort distributions and bias statistical inference.^17^, ^18^, ^19^ All analyses were performed in R (version 4.5.2) using the packages NADA2, NADA, survival, EnvStats, ggplot2, dplyr, tidyr, readxl, tibble, and stringr. Distributional characteristics were assessed using Kaplan–Meier estimators for censored data, with empirical cumulative distribution functions and group‐specific summaries calculated accordingly. Differences among forage types and sampling days were evaluated using Peto–Peto generalized Wilcoxon tests, and a two‐way censored analysis including interaction effects was applied where appropriate. Supporting analyses were conducted to evaluate robustness and included contingency analyses of detection frequencies, rank‐based tests after re‐censoring non‐detects, parametric censored analyses on the log scale, permutation tests, and interval‐censored lognormal regression models, consistent with recommended approaches for microbiological count data subject to detection limits.^20^, ^21^, ^22^

## RESULTS

### Chemical–physical parameters

Table 1 reports the effect of the different EFs and EPs, and their interaction on chemical parameters.

**Table 1:** Effect of ensiled forage and ensiling period on the chemical parameters of baled silage

Item	Units of measurement	Ensiled forage^a^				Ensiling period^b^					Model effect (*P* < 0.05)^c^			
		G	L	GM	MGL	30	60	90	180	270	SEM	EF	EP	EF*EP
*n*		19	20	25	51	26	24	20	25	20				
DM	g kg^−1^	433.62	478.62	402.64	490.21	408.73	506.64	447.51	422.19	457.19	10.701	0.72	0.09	0.59
Ash	g kg^−1^ DM	83.65	93.82	95.11	81.56	79.62	92.80	91.87	90.34	88.03	1.874	0.51	0.27	0.01
CP	g kg^−1^ DM	112.20b	166.38a	115.31b	124.14b	121.93	129.57	129.91	120.37	118.06	3.410	0.006	0.20	0.09
NDF	g kg^−1^ DM	431.94	438.10	447.66	404.38	410.25	435.50	413.82	459.77	433.25	7.923	0.87	0.37	0.72
ADF	g kg^−1^ DM	287.58	298.63	258.15	249.66	252.35	263.94	263.08	291.99	296.16	6.991	0.74	0.20	0.26
ADL	g kg^−1^ DM	33.64b	76.12a	37.53b	40.70b	49.20	46.50	45.27	46.04	47.98	1.548	0.001	0.83	0.09
EE	g kg^−1^ DM	23.59	22.20	22.91	17.61	20.94	21.38	21.36	22.63	21.57	0.490	0.17	0.66	0.70
Starch	g kg^−1^ DM	174.37	25.56	156.74	155.43	120.08	122.95	123.28	135.60	138.21	5.162	0.001	0.09	0.02

DM, dry matter; CP, crude protein; NDF, neutral detergent fiber; ADF, acid detergent fiber; ADL, acid detergent lignin; EE, ether extract. Different letters (a, b) indicate statistically significant differences (*P* < 0.05).

^a^ Ensiled forage used for making the silage: G, pure grasses; L, legumes; GM, grass mix; MGL, mix of grasses and legumes.

^b^ Ensiling period: 30, 60, 90, 180, and 270 days, respectively.

^c^ The model effect is significant for *P* < 0.05.

**Figure 1:**
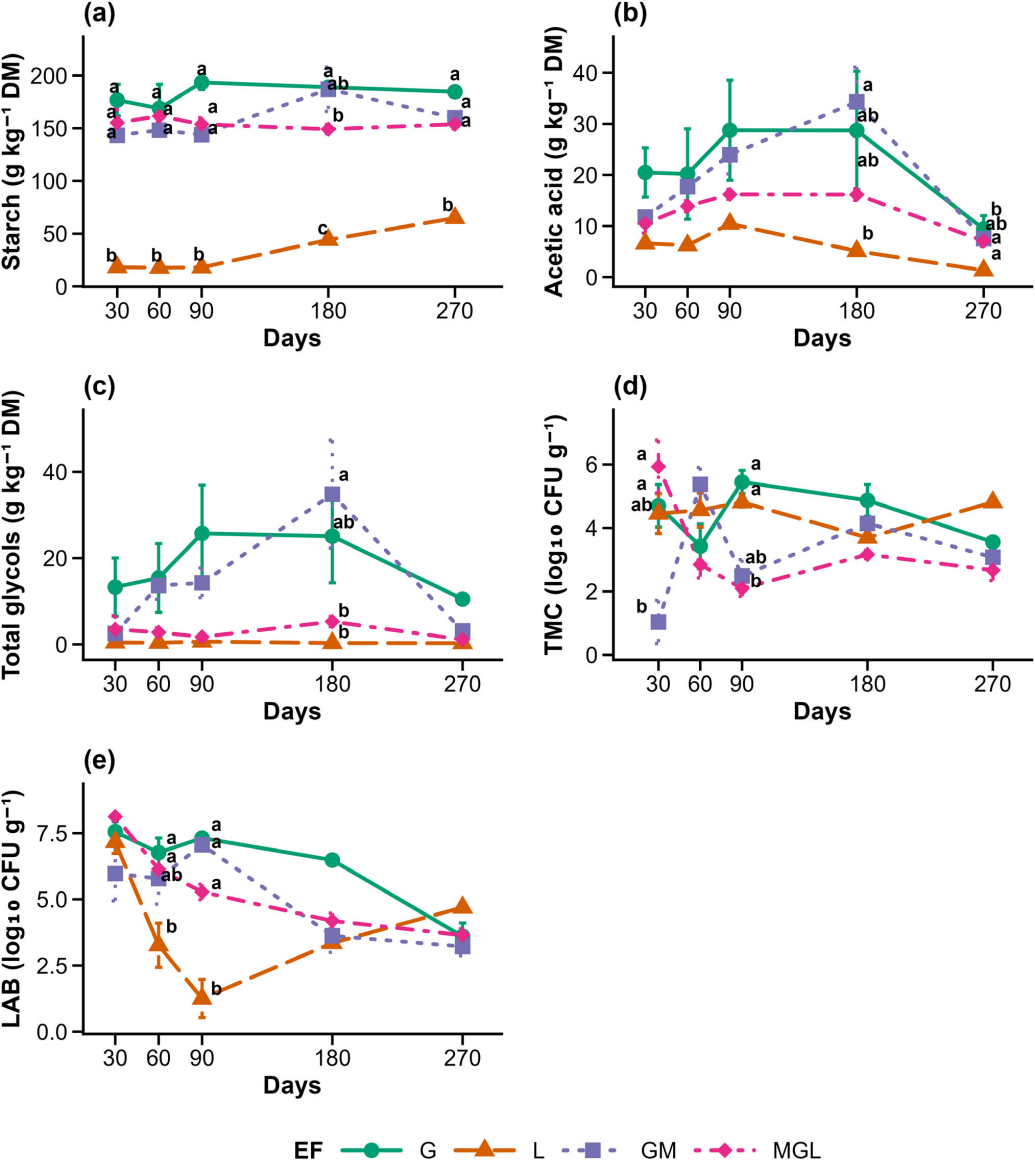
Effects of ensiling period and ensiled forage on (a) starch, (b) acetic acid, (c) total glycols, (d) total mesophilic count (TMC), and (e) lactic acid bacteria (LAB). Points represent mean values and error bars indicate the standard error. Different colors, line types, and symbols identify the ensiled forage (EF) categories: G, pure grasses; L, legumes; GM, grass mixture; and MGL, mixture of grasses and legumes. Starch and acetic acid are expressed as g kg^−1^ DM, total glycols as mg kg^−1^ DM, and microbial counts as log_10_ CFU g^−1^. Different lower‐case letters indicate significant differences among ensiling times within the same forage type according to post hoc comparisons following the mixed‐model analysis (*P* < 0.05).

The effect of EF was significant for CP (*P* = 0.006) and ADL (*P* < 0.001). The highest CP value, as expected, was observed in legume‐based bales (L; i.e., 166.38 g kg^−1^ DM), whereas the lowest for grass‐based bales (G; i.e., 112.20 g kg^−1^ DM). On the other hand, L had the highest ADL (i.e., 76.12 g kg^−1^ DM), whereas the lowest ADL value was reported for G (i.e., 33.64 g kg^−1^ DM). Although the overall test for EF was significant for starch, its interpretation was conditioned by the significant EF × EP interaction (*P* = 0.020). Therefore, starch dynamics were interpreted through the interaction plot shown in Fig. 1(a). Starch concentrations were generally similar among forage types, with the exception of L, which showed the lowest value (i.e., 25.56 g kg^−1^ DM). The effect of EP was not significant for any of the analyzed parameters. However, a significant interaction between EF and EP (*P* = 0.020) was observed for starch concentration. G and MGL showed relatively stable starch concentrations throughout the ensiling period, whereas GM exhibited a peak at 180 days, followed by a slight decrease at 270 days. By contrast, L showed a progressive increase over time, with the lowest values at 30–90 days and higher concentrations at 180 and 270 days, although still remaining lower than those of the other forage types.

### Fermentative parameters

The effects of EF, EP, and their interaction on fermentative parameters are reported in Table 2. Propionic acid was significantly affected by both main effects, namely EF and EP. The highest propionic acid concentration was observed in MGL‐based bales (0.10 g kg^−1^ DM), whereas the lowest was found in G‐based bales (0.08 g kg^−1^ DM). In addition, propionic acid concentration significantly decreased by 66.5% from 180 to 270 days. However, despite the statistical significance, the absolute difference was very small and is therefore likely of limited biological relevance.

**Table 2:** Effect of ensiled forage and ensiling period on the main volatile organic compounds of baled silages

Item	Units of measurement	Ensiled forage^a^				Ensiling period^b^					Model effect (*P* < 0.05)^c^			
		G	L	GM	MGL	30	60	90	180	270	SEM	EF	EP	EF*EP
*n*		19	20	25	51	26	24	20	25	20				
pH	dmnl	4.64	4.74	4.13	4.50	4.39	4.61	4.44	4.47	4.54	0.049	0.65	0.72	0.64
Ammonia N	g kg^−1^ TKN	40.54	23.01	41.51	28.15	17.82b	23.90b	26.42b	30.79b	69.51a	2.849	0.20	0.001	0.94
Lactic acid	g kg^−1^ DM	26.21	9.57	52.97	38.74	50.70a	27.45ab	33.85ab	27.61ab	19.75b	1.999	0.193	0.010	0.113
Acetic acid	g kg^−1^ DM	11.39	9.59	19.31	12.79	11.94	13.83	18.20	19.88	2.51	1.055	0.746	<0.001	0.001
Propionic acid	g kg^−1^ DM	0.08a	0.05ab	0.07ab	0.10b	0.08a	0.08a	0.09a	0.09a	0.03b	0.004	0.028	<0.001	0.129
1,2‐Propanediol	g kg^−1^ DM	1.54	4.91	10.97	7.85	4.22b	3.56b	14.85a	6.26b	2.69b	1.250	0.451	0.030	0.646
Ethanol	g kg^−1^ DM	6.87	2.50	5.05	5.83	5.32	3.67	4.83	6.78	4.70	0.604	0.514	0.805	0.577
Methanol	g kg^−1^ DM	0.57	1.56	0.91	0.98	0.94ab	1.22a	1.47a	1.11ab	0.29b	0.097	0.301	0.002	0.051
2,3‐Butanediol	mg kg^−1^ DM	22.11	37.95	40.16	51.18	27.25	60.85	53.61	23.56	23.99	7.396	0.640	0.599	0.803
Acetaldehyde	mg kg^−1^ DM	91.42	98.20	105.7	67.74	119.2 a	92.16a	101.9a	116.4a	28.6b	5.384	0.460	<0.001	0.142
Ethyl acetate	mg kg^−1^ DM	21.40	5.25	48.58	14.80	27.03	22.18	15.37	40.37	16.35	2.723	0.311	0.137	0.803
Ethyl lactate	mg kg^−1^ DM	34.96	12.86	43.63	46.21	15.24b	22.32b	45.34ab	66.28a	36.25ab	4.051	0.475	0.030	0.867
Total aldehydes	mg kg^−1^ DM	141.19	164.47	201.68	112.27	205.77ab	112.23bc	158.23abc	228.80a	69.49c	10.141	0.421	<0.001	0.255
Total esters	mg kg^−1^ DM	88.71	31.94	106.9	106.6	91.23	68.81	86.76	98.15	72.79	9.739	0.240	0.950	0.729
Total ketones	mg kg^−1^ DM	11.44	10.82	21.01	12.48	18.52	16.75	2.26	28.33	3.83	4.287	0.937	0.573	0.395
Total glycols	mg kg^−1^ DM	579	398	1393	279	588	779	773	1268	326	125.548	<0.001	0.691	0.001
acLa/acAc	dmnl	2.66	2.90	3.62	3.81	4.30ab	2.23ab	2.20ab	1.68b	5.83a	0.362	0.888	0.017	0.932
acLa/acAc+EtOH	dmnl	2.43	2.73	3.47	3.63	4.11ab	2.15ab	2.10ab	1.58b	5.39a	0.335	0.865	0.017	0.936

Ammonia N: ammonia nitrogen expressed on a total N (TKN) basis; dmnl, dimensionless. Different letters (a–c) indicate statistically significant differences (*P* < 0.05). acLa/acAc: lactic acid/acetic acid ratio; acLa/acAc+EtOH: lactic acid/acetic acid+ethanol ratio.

^a^ Ensiled forage used for making the silage: G, pure grasses; L, legumes; GM, grass mix; MGL, mix of grasses and legumes.

^b^ Period: 30, 60, 90, 180, and 270 days, respectively.

^c^ The model effect is significant for *P* < 0.05.

The pH remained almost constant throughout the storage period, ranging from 4.39 at 30 days to 4.54 at 270 days. Ammonia N increased (*P* < 0.001) with the advance of EL, rising from 17.82 g kg^−1^ TKN at 30 days to 69.51 g kg^−1^ TKN at 270 days. Lactic acid decreased (*P* = 0.010) over the storage period, from a concentration of 50.70 g kg^−1^ DM at day 30 to 19.75 g kg^−1^ DM at day 270.

Interaction between EF and EP showed a significant effect on acetic acid (*P* < 0.001). Its concentration increased up to day 180 (19.88 g kg^−1^ DM), followed by a decrease at day 270 (2.51 g kg^−1^ DM), as shown in Fig. 1(b). This increase was more pronounced in the MG‐based baled silage, which reached 19.31 g kg^−1^ DM. The concentration of 1,2‐propanediol increased (*P* = 0.030) by 71.6% from 30 to 90 days; notably, the highest value of 1,2‐propanediol was detected at 90 days (i.e., 14.85 g kg^−1^ DM). Methanol was affected (*P* = 0.002) by EP and was higher in bales sampled at 90 days than 270 days (i.e., 1.47 *vs*. 0.29 g kg^−1^ DM, respectively). The concentration of total aldehydes was influenced (*P* < 0.001) by EP, reaching the highest value at 180 days (i.e., 228.80 mg kg^−1^ DM) and the lowest at 270 days (i.e., 69.49 mg kg^−1^ DM). Acetaldehyde decreased (*P* < 0.001) by 76.0% from 30 to 270 days. Ethyl lactate increased (*P* = 0.030) with the advance of EP, reaching a peak at 180 days (i.e., 66.28 mg kg^−1^ DM). The interaction between EF and EP had a significant effect on total glycols (Fig. 1(c), *P* = 0.001). Their concentration increased up to day 180 (1268 mg kg^−1^ DM), followed by a decrease at day 270 (326 mg kg^−1^ DM). The highest concentration was observed in the MG‐based baled silage.

As for the other volatile compounds, no significant effects were observed in relation to either ensiled forage or ensiling period, as shown in Table 2.

### Microbiological analyses

The effect of EF, EP and their interaction on microbiological composition of baled silages are reported in Table 3. TMC counts were significantly influenced by the EP × EF interaction (*P* < 0.001), showing distinct time‐related patterns among silage types (Fig. 1(e)). Legume silage showed a comparatively more stable profile over time, with only moderate fluctuations, whereas GM silage displayed the most variable pattern, characterized by marked oscillations across sampling times. By contrast, MGL silage exhibited the highest TMC at 30 days, followed by a sharp reduction and persistently lower values during prolonged storage, suggesting a more rapid microbiological stabilization after the initial fermentation phase. LAB counts were also significantly affected by the EP × EF interaction (*P* < 0.001), indicating that their temporal evolution depended on silage type (Fig. 1(e)). In G silage, TMC decreased at 60 days, peaked at 90 days, and then progressively declined towards 270 days. L silage displayed a comparatively more stable pattern over time, with only moderate fluctuations and a slight increase at the end of storage. GM silage showed marked oscillations, with low counts at 30 days, a sharp increase at 60 days, a decrease at 90 days, and intermediate values thereafter. By contrast, MGL silage had the highest TMC count at 30 days, followed by a pronounced decline and persistently lower counts at later sampling times. Overall, LAB populations tended to decline during prolonged storage, but the magnitude and timing of this decrease differed among silages. In G silage, LAB counts remained relatively high up to 180 days and then dropped markedly at 270 days. In contrast, L silage showed the sharpest reduction already at 60–90 days, followed by a partial recovery at later storage times. GM silage showed a transient increase at 90 days and then declined thereafter, whereas MGL silage exhibited a more progressive decrease over the storage period.

**Table 3:** Effect of ensiled forage and ensiling period on the main microbial groups of baled silages

Item	Units of measurement	Ensiled forage^a^				Ensiling period^b^				Model effect (*P* < 0.05)^c^			
		G	L	GM	MGL	30	60	90	180	270	SEM	EF	EP	EF*EP
*n*		19	20	25	51	26	24	20	25	20				
TMC	log_10_ CFU g^−1^	5.00	5.10	3.22	3.33	4.10	4.50	4.08	4.10	4.03	0.122	0.396	0.798	<0.001
LAB	log_10_ CFU g^−1^	5.78	3.27	5.13	5.40	7.19	5.38	4,88	3.86	3.17	0.134	0.108	<0.001	<0.001
ASFB	log_10_ CFU g^−1^	3.21a	3.49a	3.43a	2.8b	3.07	3.23	3.08	3.56	3.22	0.071	0.003	0.456	0.782

TMC, total mesophilic count; LAB, lactic acid bacteria; ASFB, aerobic spore‐forming bacteria. Different letters (a, b) indicate statistically significant differences (*P* < 0.05).

^a^ Ensiled forage used for make the silage: G, pure grasses; L, legumes; GM, grass mix; MGL, mix of grasses and legumes.

^b^ Ensiling period: 30, 60, 90, 180, and 270 days, respectively.

^c^ The model effect is significant for *P* < 0.05.

**Figure 2:**
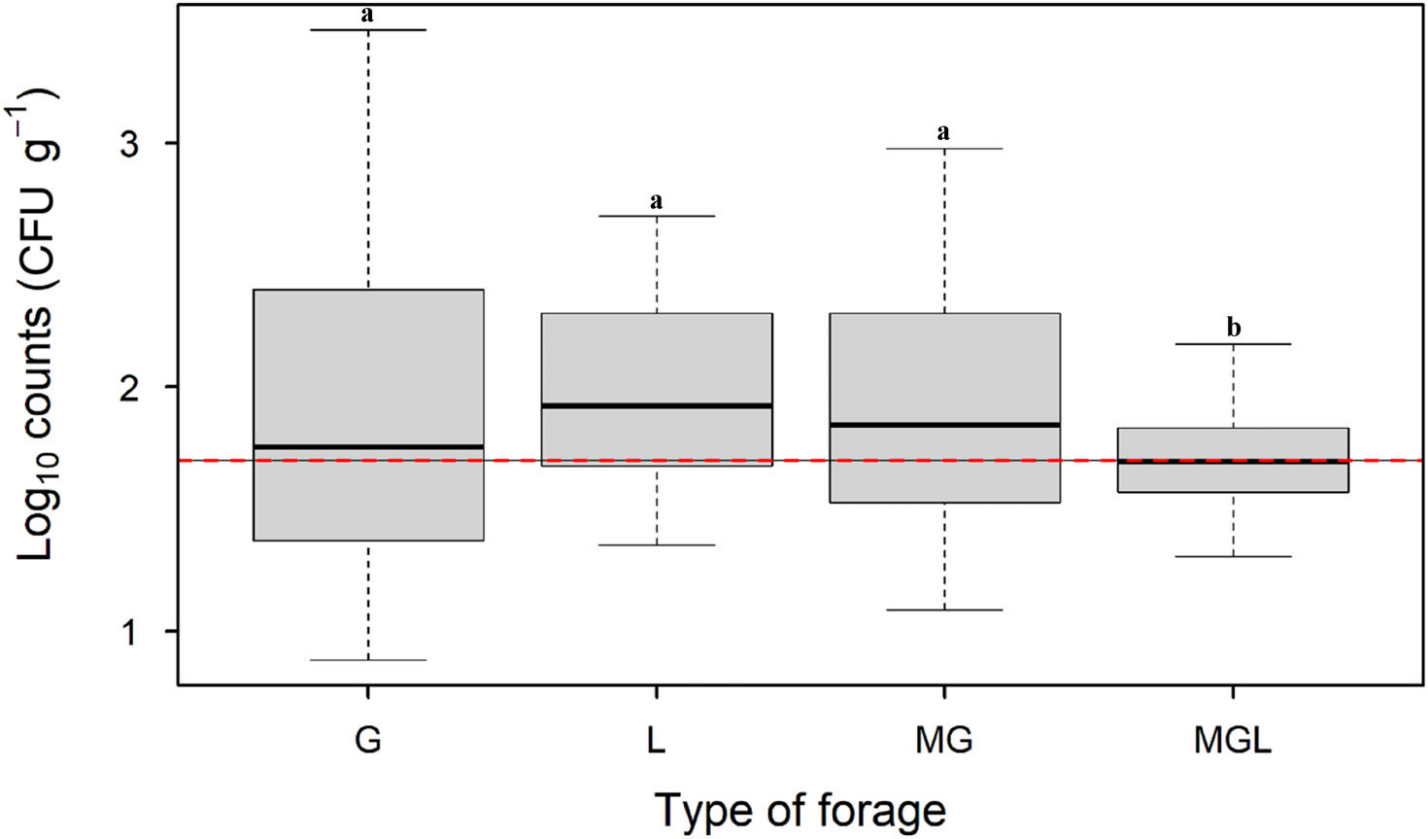
Censored boxplot of clostridia counts by forage type. Data are expressed as log_10_ CFU g^−1^ and analyzed as left‐censored observations. Boxes represent Kaplan–Meier‐based censored distributions, and the horizontal dashed line indicates the detection limit (LOD = 50 CFU g^−1^). G, pure grasses; L, legumes; GM, grass mixture; and MGL, mixture of grasses and legumes. Different superscript letters indicate statistically significant differences (*P* < 0.001).

Considering the effect of EF, differences for the count of ASFB were observed (*P* = 0.003). The MGL‐based bales had the lowest value of ASFB (i.e., 2.8 log_10_ CFU g^−1^). Y&M counts were characterized by a high proportion of left‐censored observations across all sampling days (ranging from 70% to 84% < LOD) and forage types (ranging from 72% to 86% < LOD), indicating generally low levels of contamination throughout the experimental period. Kaplan–Meier‐based censored boxplots showed largely overlapping distributions among sampling days, with no consistent temporal trend and only sporadic high detected values (Supporting Information, Fig. S1). Consistently, no significant differences among sampling days were detected by Peto–Peto tests (*P* = 0.22). Similarly, censored distributions overlapped across forage types, and no statistically significant differences were observed among groups (*P* = 0.47), despite higher dispersion in some ensiled forage in MGL type (Supporting Information, Fig. S2). In legume silage the median log_10_ CFU g^−1^ counts were above the LOD with respect to the other ensiled forage. Two‐way censored analysis including forage type and sampling day confirmed the absence of strong main effects, suggesting that observed variability was driven by isolated high counts rather than systematic group‐level shifts. Visualization of individual observations further highlighted the predominance of non‐detects and the limited influence of detected values on overall distributional patterns (Supporting Information, Figs S3 and S4). Overall, clostridia counts were low across all baled silages, especially in MGL‐based bales (i.e., 0.40 log_10_ CFU g^−1^). Clostridia counts were extensively left‐censored, with approximately 73% of observations below the detection limit (LOD = 50 CFU g^−1^). Kaplan–Meier‐based analyses showed no effect of sampling day on clostridia distributions (*P* = 0.68; Supporting Information, Fig. S5), indicating the absence of consistent temporal trends during storage. In contrast, a significant effect of forage type was detected (*P* < 0.001), with pairwise comparisons showing that MGL consistently exhibited lower clostridia levels than G, L, and MG, which did not differ from each other (Fig. 2). Two‐way censored modeling confirmed forage type as the sole relevant explanatory factor. Visualization of individual observations supported these findings, highlighting the predominance of non‐detects in MGL and the higher dispersion observed in the other forage types, while temporal patterns remained weak and inconsistent (Supporting Information, Figs S6 and S7). No presence of *Escherichia coli* or *Listeria monocytogenes* was detected in the different baled silages. In some samples, typical colonies appeared on the enumeration plates; however, molecular analysis revealed that they were not *Listeria monocytogenes* and represented false positives. Further analyses are underway to identify these isolates at the species level.

## DISCUSSION

### Chemical composition of baled silage during storage

Round bale ensiling represents a widely established conservation technique for maintaining forage quality, offering an effective option for preserving the high nutritional value of early cut grass.^1^, ^23^, ^24^ The amount of total organic acids in the mass is strongly related to moisture content, which influenced the development of microorganisms. The moisture content in baled silage tends to be lower than in conventional silage stored in horizontal silos.^24^, ^25^ The DM content in the analyzed samples of baled silage was, on average, consistent with typical values reported for this type of product.^2^ However, some variability in DM values was observed, likely reflecting differences in forage type, the extent of pre‐ensiling wilting, and harvesting and baling management, rather than harvest maturity alone. The NDF was lower than values reported by other authors for grass‐based wrapped bales,^1^, ^26^ reflecting the higher nutritional value of the forage. Only 7.5% of the samples had an NDF content greater than 50% on a DM basis. Although protein content was variable, a high average level of CP was observed, with concentrations exceeding 10% in 76% of the samples. Taken together, the relatively low NDF and high CP contents suggest that a substantial proportion of the forage was harvested at a relatively early stage of maturity and/or included mixed forage species with higher nutritional value. However, this interpretation is supported primarily by the chemical composition rather than by DM content itself.

### Effect on fermentative parameters

The pH is considered the main indicator of silage quality and the fermentation process, which is essential for obtaining high‐quality silage. Only 9% of the analyzed samples showed a pH > 4.85, which is considered the critical threshold for silage with 45% DM to maintain acceptable aerobic stability.^9^ In this study, pH values were positively correlated with DM^9^ and were within the typical range for this type of product, indicating appropriate fermentation and confirming silage quality. The low pH values found in most of the analyzed samples reflected the high lactic acid content, which exceeded 3% in 60% of the samples. It was associated with a low concentration of butyric acid, and low ammonia N content, the last rarely exceeding 12% – a threshold beyond which silage may negatively affect intake and digestibility when included in animal rations.^27^, ^28^ Lactic acid tended to decrease significantly during the storage period, likely due to its metabolism by heterofermentative bacteria,^28^ mainly producing acetic acid and 1,2‐propanediol, which increased over time. These compounds are believed to be critical for aerobic stability. Acetic acid, known to enhance aerobic stability, may also be further metabolized by bacteria when other substrates become limited. The concentration of 1,2‐propanediol followed a trend similar to that of acetic acid, likely reflecting the activity of heterofermentative LAB converting lactic acid into these compounds.^28^ The metabolism of lactic acid during the storage period is further confirmed by the acLa/acAc and acLa/acAc+EtOH ratios, both of which were significantly influenced by the ensiling period.^29^ Methanol concentration increased until 90 days and then decreased until 270 days (Table 2). It should be noted that methanol is derived from pectin metabolism; in fact, methanol concentration was numerically higher in legume silages.^30^, ^31^, ^32^ Pectins are nonfibrous carbohydrates that make up about 10–12% of the carbohydrates in the cell wall of alfalfa stems. Generally, legumes contain more pectins than grasses.^33^ In agreement with other authors, pectins present in legumes may play an important role in modulating microbial fermentation in silage, particularly in alfalfa silage.^34^ The concentration of total aldehydes increased notably up to 180 days of ensiling, followed by a sharp decrease at 270 days. These volatile compounds may influence animal feeding preferences, palatability, and intake.^35^, ^36^ Furthermore, the potential transfer of these compounds to milk and meat and their impact on sensory properties has also been considered.^37^

### Effect on microbiological count

The analyzed baled silage had a good microbiological quality, with a low count of spoilage microorganisms. In particular, yeast and clostridia populations were found to be very low. These results demonstrate the excellent microbiological quality of most of the samples of baled silage analyzed. Indeed, the number of clostridial spores was quite low. According to the classification of silage contamination levels,^38^ defined as low when spore counts are below 3 log_10_ CFU g^−1^, moderate between 3 and 5 log_10_ CFU g^−1^, and high when exceeding 5 log_10_ CFU g^−1^, nearly all the baled silage samples analyzed fall within the low contamination category. As for LAB, as expected, they were present in high numbers compared to the other microbial groups investigated. ASFB were present in very low numbers, suggesting the adoption of good management practices for wrapped silage hay. This is consistent with previous studies highlighting how appropriate silage management helps maintain low spore counts.^35^ Regarding the presence of pathogens in the haylage samples used, *Escherichia coli* and *Listeria monocytogenes* were not found in the baled silage samples analyzed. This result is particularly significant considering the potential risk posed by these microorganisms along the dairy supply chain. With regard to *Listeria monocytogenes*, colonies showing the typical morphology of the species were isolated from ALOA plate; in the first instance, the samples were considered contaminated with *Listeria monocytogenes*; however, molecular analysis yielded negative results, as no amplicons were obtained using species‐specific PCR. Forage contamination by these pathogens primarily results from the application of manure or other organic soil amendments in the field, or through contact with contaminated soil particles during harvesting.^39^ The high microbiological quality observed in the baled silage samples in the present study is consistent with the findings of Borreani *et al*.,^40^ who reported that haylage obtained from earlier‐cut permanent meadows improved forage quality without increasing the risk of clostridial contamination from forage to milk and cheese. Notably, those authors found no detrimental effects on cheese quality after ripening, indicating that the use of conserved forages with higher moisture content is not necessarily associated with poorer hygienic or technological outcomes. In this context, our results support the view that wrapped baled forage, when properly produced and managed, may represent a safe conservation strategy even in dairy systems where silage is still regarded with caution. Therefore, our study may be considered a first step toward reassessing the risks attributed to baled silage in sheep farming systems and, more broadly, in dairy areas where its adoption remains limited because of concerns about cheese quality.

## CONCLUSIONS

Long‐term storage up to 270 days did not impair the chemical–physical or hygienic quality of baled silage. pH remained stable throughout storage, while fermentation end‐products evolved as expected, with declining lactic acid and time‐dependent changes in acetic acid and 1,2‐propanediol, alongside an increase in ammonia N. Spoilage microorganisms remained low, with yeasts and molds and clostridia largely below detection limits, while *Escherichia coli* and *Listeria monocytogenes* were not detected in any sample. Overall, these findings support the suitability of baled silage for prolonged conservation under Mediterranean farm conditions.

## FUNDING INFORMATION

This work was carried out within the framework of the project *e.INS – Ecosystem of Innovation for Next Generation Sardinia* (code ECS00000038), funded by the Italian Ministry for University and Research (MUR) under the National Recovery and Resilience Plan (NRRP) – Mission 4, Component 2, ‘From Research to Business,’ Investment 1.5, ‘Creation and strengthening of innovation ecosystems’ and the construction of ‘Territorial R&D Leaders.’

This publication, “Physicochemical properties, fermentative profile, microbiological quality, and pathogen contamination of baled silage during long‐term storage,” was produced while Lidia Nieddu was attending the PhD programme in Sustainable Development and Climate Change at the University School for Advanced Studies IUSS Pavia, Cycle XL, with the support of a scholarship co‐financed by the Italian Ministry of University and Research (MUR) under Ministerial Decree No. 629 of 24 April 2024, within the National Recovery and Resilience Plan (NRRP), financed by the European Union – NextGenerationEU, Mission 4 “Education and Research,” Component 1 “Enhancement of the offer of educational services: from nurseries to universities,” Investment 3.4 “Advanced university teaching and skills” and Investment 4.1 “Extension of the number of research doctorates and innovative doctorates for public administration and cultural heritage.

## AUTHOR CONTRIBUTIONS

Lidia Nieddu: writing – review and editing; writing – original draft; investigation; data curation. Mirko Fresu: investigation. Carmelo Mastroeni: writing – review and editing; investigation; data curation. Antonio Gallo: methodology; writing – review and editing; data curation. Severino Zara: writing – review and editing; funding acquisition. Francesco Fancello: writing – review and editing; writing – original draft; supervision; conceptualization; methodology; formal analysis; validation; data curation; resources. Alberto Stanislao Atzori: supervision; conceptualization; methodology; validation; resources; funding acquisition.

## Supporting information


**Figure S1.** Censored boxplot of yeast and mold (Y&M) counts by sampling day. Data are expressed as log_10_ CFU g^−1^ and analyzed as left‐censored observations. Boxes represent Kaplan–Meier‐based censored distributions, and the horizontal dashed line indicates the detection limit (LOD = 100 CFU g^−1^).

**Figure S2.** Censored boxplot of yeast and mold (Y&M) counts by forage type. Data are expressed as log_10_ CFU g^−1^ and analyzed as left‐censored observations. Boxes represent Kaplan–Meier‐based censored distributions, and the horizontal dashed line indicates the detection limit (LOD = 100 CFU g^−1^).

**Figure S3.** Distribution of yeast and mold (Y&M) counts by sampling day with individual observations overlaid. Data are expressed as log_10_ CFU g^−1^; non‐detects (<LOD) are plotted at half the detection limit for visualization purposes only, while statistical analyses were performed using appropriate censored‐data methods. The horizontal dashed line indicates the detection limit (LOD = 100 CFU g^−1^).

**Figure S4.** Distribution of yeast and mold (Y&M) counts by forage type with individual observations overlaid. Data are expressed as log_10_ CFU g^−1^; non‐detects (<LOD) are plotted at half the detection limit for visualization purposes only, while statistical analyses were performed using appropriate censored‐data methods. The horizontal dashed line indicates the detection limit (LOD = 100 CFU g^−1^).

**Figure S5.** Censored boxplot of clostridia counts by sampling day. Data are expressed as log_10_ CFU g^−1^ and analyzed as left‐censored observations. Boxes represent Kaplan–Meier‐based censored distributions, and the horizontal dashed line indicates the detection limit (LOD = 50 CFU g^−1^).

**Figure S6.** Distribution of clostridia counts by sampling day with individual observations overlaid. Data are expressed as log_10_ CFU g^−1^; non‐detects (<LOD) are plotted at half the detection limit for visualization purposes only, while statistical analyses were performed using appropriate censored‐data methods. The horizontal dashed line indicates the detection limit (LOD = 50 CFU g^−1^).

**Figure S7.** Distribution of clostridia counts by forage type with individual observations overlaid. Data are expressed as log_10_ CFU g^−1^; non‐detects (<LOD) are plotted at half the detection limit for visualization purposes only, while statistical analyses were performed using appropriate censored‐data methods. The horizontal dashed line indicates the detection limit (LOD = 50 CFU g^−1^).

**Table S1.** Botanical composition of forage types and number of plastic film layers.

**Table S2.** Calibration performances of the NIR system for the analyzed parameters.

## Data Availability

The data that support the findings of this study are available on request from the corresponding author. The data are not publicly available due to privacy or ethical restrictions.
