# Supplementary material for: Physicochemical properties, fermentative profile, microbiological quality, and pathogen contamination of baled silage during long‐term storage
Source: J Sci Food Agric. 2026 Jun 25;106(12):7454–63. doi: 10.1002/jsfa.70807 (PMC13442709; doi:10.1002/jsfa.70807)
Supplement: Supplementary file 1 [file JSFA-106-7454-s001.docx]

**Censored boxplot of yeasts and moulds (Y&M) counts by forage type**

**Supplementary Fig. 2.** Censored boxplot of yeasts and moulds (Y&M) counts by forage type. Data are expressed as log₁₀ cfu g^-1^ and analysed as left-censored observations. Boxes represent Kaplan–Meier–based censored distributions, and the horizontal dashed line indicates the detection limit (LOD= 100 cfu g^-1^).
