# Supplementary material for: Physicochemical properties, fermentative profile, microbiological quality, and pathogen contamination of baled silage during long‐term storage
Source: J Sci Food Agric. 2026 Jun 25;106(12):7454–63. doi: 10.1002/jsfa.70807 (PMC13442709; doi:10.1002/jsfa.70807)
Supplement: Supplementary file 2 [file JSFA-106-7454-s002.docx]

**Distribution of Clostridia counts by sampling day with individual observations overlaid**.

**Supplementary Fig. 6.** Distribution of Clostridia counts by sampling day with individual observations overlaid. Data are expressed as log₁₀ cfu g^-1^; non-detects (<LOD) are plotted at half the detection limit for visualization purposes only, while statistical analyses were performed using proper censored-data methods. The horizontal dashed line indicates the detection limit (LOD = 50 cfu g^-1^).
