# Supplementary material for: Physicochemical properties, fermentative profile, microbiological quality, and pathogen contamination of baled silage during long‐term storage
Source: J Sci Food Agric. 2026 Jun 25;106(12):7454–63. doi: 10.1002/jsfa.70807 (PMC13442709; doi:10.1002/jsfa.70807)
Supplement: Supplementary file 3 [file JSFA-106-7454-s003.docx]

**Supplementary Table S1.** Botanical composition of forage types and number of plastic film layers

| **Type of Forage** | **Species** | **Number of plastic film layers** |
| --- | --- | --- |
| G | Ryegrass or Barley | 8/10 |
| L | Alfalfa, Different types of Clover (*Trifolium michelianum, Trifolium subterraneum*,), | 8/10 |
| MG | Tetraploid ryegrass + Oat | 8/10 |
| MGL | Tetraploid ryegrass + Clover (*Trifolium michelianum, Trifolium subterraneum* ) | 8/10 |

G: pure grasses; L: legumes, MG: grass mix; MGL: mix of grasses and legumes.
