# Supplementary material for: Physicochemical properties, fermentative profile, microbiological quality, and pathogen contamination of baled silage during long‐term storage
Source: J Sci Food Agric. 2026 Jun 25;106(12):7454–63. doi: 10.1002/jsfa.70807 (PMC13442709; doi:10.1002/jsfa.70807)
Supplement: Supplementary file 5 [file JSFA-106-7454-s005.docx]

**Supplementary Table S2.** Calibration performances of the NIR system for the analyzed parameters.

| **Parameter** | **SECV^1^** | **1V-R^2^** |
| --- | --- | --- |
| Moisture | 2.30 | 0.97 |
| CP | 0.78 | 0.95 |
| EE | 0.21 | 0.40 |
| Fiber | 1.69 | 0.83 |
| Ash | 1.02 | 0.73 |
| Starch | 1.29 | 0.53 |
| NDF | 2.67 | 0.86 |
| ADF | 2.61 | 0.78 |
| ADL | 0.40 | 0.67 |

^1^ SECV = standard error of cross-validation.

^2^ 1V-R^2^ = coefficient of determination.

CP: Crude Protein; NDF: Neutral Detergent Fiber; ADF: Acid Detergent Fiber; ADL: Acid Detergent Lignin; EE: Ether Extract.
